# Development of a One-Step Probe Based Molecular Assay for Rapid Immunodiagnosis of Infection with *M. tuberculosis* Using Dried Blood Spots

**DOI:** 10.1371/journal.pone.0105628

**Published:** 2014-09-03

**Authors:** Thomas Blauenfeldt, Jan Heyckendorf, Sidse Graff Jensen, Christoph Lange, Camilla Drabe, Thomas S. Hermansen, Lena de Thurah, Troels Lillebaek, Jesper Eugen-Olsen, Niels Seersholm, Søren Hoff, Jesper Bonde, Morten Ruhwald

**Affiliations:** 1 Department of Infectious Disease Immunology, Statens Serum Institut, Copenhagen, Denmark; 2 Clinical Infectious Diseases, German Center for Infection Research (DZIF) Tuberculosis Unit, Research Center Borstel, Borstel, Germany; 3 Department of Pulmonary Medicine, Copenhagen University Hospital Gentofte, Copenhagen, Denmark; 4 International Reference Laboratory of Mycobacteriology, Statens Serum Institut, Copenhagen, Denmark; 5 Clinical Research Centre, Copenhagen University Hospital Hvidovre, Copenhagen, Denmark; 6 Department of Pathology, Copenhagen University Hospital Hvidovre, Copenhagen, Denmark; 7 Department of Medicine, University of Namibia School of Medicine, Windhoek, Namibia; University of Cape Town, South Africa

## Abstract

**Background:**

Antigen specific release of IP-10 is the most promising alternative marker to IFN-γ for infection with *M. tuberculosis*. Compared to Interferon-γ release assays (IGRA), IP-10 is released in high levels enabling novel approaches such as field friendly dried blood spots (DBS) and molecular detection.

**Aim:**

To develop a robust IP-10 based molecular assay for the diagnosis of infection with *M. tubercuolsis* from whole blood and DBS.

**Method:**

We developed a one-step probe based multiplex RT-qPCR assay for detecting IP-10 and IFN-γ mRNA expression from whole blood and DBS samples. The assay was validated and applied for the diagnosis of *M. tuberculosis* infection in DBS samples from 43 patients with confirmed TB, 13 patients with latent TB and 96 presumed uninfected controls. In parallel, IP-10 and INF-γ levels were measured in Quantiferon (QFT-TB) plasma supernatants.

**Results:**

IP-10 mRNA upregulation was detectable at 4 hours after stimulation (6 fold upregulation) peaking at 8 hours (108 fold upregulation). IFN-γ expression occurred in concert but levels were lower (peak 6.7 fold upregulation). IP-10 gene expression level was significantly higher in patients with tuberculosis (median 31.2, IQR 10.7–67.0) and persons with latent tuberculosis infection (LTBI) (41.2, IQR 9.8–64.9) compared to healthy controls (1.6, IQR 1.1–2.4; p<0.0001). The IP-10 mRNA and protein based tests had comparable diagnostic accuracy to QFT-TB, sensitivity (85% and 88% vs 85%) and specificity (96% and 96% vs 97%, p = ns.).

**Conclusion:**

We developed a rapid, robust and accurate molecular immunodiagnostic test for *M. tuberculosis* infection. By combining DBS based sample acquisition, mail or currier based sample transport with centralized molecular detection, this immunodiagnostic test concept can reduce the local technological requirements everywhere and make it possible to offer highly accurate immunodiagnostic tests in low resource settings.

## Introduction

Tuberculosis (TB) remains a leading cause of morbidity and mortality worldwide with approximately 8.6 million new cases and 1.3 million deaths in the year 2012 [1]. In the absence of “active” tuberculosis an estimated 2 billion people have a detectable immune response towards *M. tuberculosis*, the causative agent of tuberculosis. Although infection cannot be directly proven in this situation, presence of an adaptive immune response to antigens that are relatively specific for *M. tuberculosis* is defined as latent infection with *M. tuberculosis* (LTBI) in the absence of disease [2]. Depending on the risk of previous exposure to *M. tuberculosis* and the immune status of the person, individuals with LTBI have a variable risk for the progression to tuberculosis [3], [4].

For almost one century, the diagnosis of LTBI has been based on the tuberculin skin test (TST) [5]. A decade ago, the *in-vitro* alternative IFN-γ release assays (IGRAs) were introduced. As implied in the name, IGRAs measure IFN-γ released after stimulation with *M. tuberculosis* specific antigens ESAT-6, CFP10, and for the whole blood based Quantiferon Gold In-Tube (QFT-TB, Qiagen, Hilden, Germany) also a single peptide from TB7.7 [6]. IGRAs are not affected by previous *M. bovis* Bacille Calmette-Guérin vaccination or exposure to the vast majority of non-tuberculous mycobacteria wherefore IGRAs provide a more specific measure of putative infection with *M. tuberculosis* than the TST [7]. However, recent reports have shown that IGRAs only perform marginally better than TST for prediction of later progression to active tuberculosis [8]–[11].

Alternative readout biomarkers to IFN-γ as predictors of the future development of tuberculosis are currently being investigated [12]. Of several candidates, interferon-γ inducible protein (IP)-10 is the most extensively investigated and most promising candidate marker at present (reviewed in [13]). IP-10 is a chemokine secreted by antigen presenting cells upon interaction with T cells recognizing its specific peptide signature presented on the major histocompatibility complex molecules. IP-10 is considered an inducible chemokine and strong upregulation has been described following stimulation with IFN-γ, TNF-α and other pro-inflammatory cytokines [14]–[16]. To date, the diagnostic performance of a protein based IP-10 test for *M. tuberculosis* infection has been evaluated in more than 40 clinical studies; showing comparable sensitivity to IFN-γ for tuberculosis cases; comparable specificity in unexposed controls; stronger association between test positivity and exposure in persons at risk of tuberculosis; better test sensitivity in HIV-infected patients with low CD4 T cell count; and more robustness in children <5 years of age [12]. However, the positive predictive value for the development of tuberculosis in individuals with a positive test result has not yet been determined for IP-10.

Compared to IFN-γ, IP-10 has been shown to be released at 100 fold higher levels following stimulation with antigens specific for *M. tuberculosis* [17] which allows for the use of simpler detection methods such as dried blood spots (DBS) and lateral flow (reviewed in [12]). Moreover, IP-10 can be detected and quantified by molecular methods at the gene expression levels using Reverse Transcription Quantitative PCR (RT-qPCR). Amplification of nucleic acids is a powerful tool for sensitive detection of transcriptional changes in low sample volumes [18]. Also, it is a well proven diagnostic approach and has shown promise for the diagnosis of *M. tuberculosis* specific immune responses using mRNA encoding IFN-γ, IL-2 and other cytokines, but in particular IP-10 [15], [19]. Kinetic studies of IFN-γ gene expression suggests that the shorter incubation is vastly superior for diagnostic assays [20], but no detailed investigations have been attempted with IP-10 possibly having led to an underestimation of the potential of the technology.

Here, we assessed the kinetics of IP-10 gene expression in response to *M. tuberculosis* specific antigen stimulation to explore the immunodiagnostic potential of mRNA detection from dried blood spots, a technique that holds great potential for the diagnosis of LTBI in resource limited settings.

## Materials and Methods

### Patients and controls

Following approval by the ethical review board of the University Lübeck, Germany (reference number 11-072, 17.5.2011) and the ethical review board of the Capital Region of Copenhagen (journal number H-3-2012-008) we included patients with pulmonary tuberculosis from the Medical Clinic of the Research Center Borstel, Borstel, Germany and the Department of Respiratory Medicine, Copenhagen University Hospitals, Gentofte, Denmark, respectively. Tuberculosis was confirmed in all patients by detection of *M. tuberculosis* from sputum or bronchopulmonary culture specimens. In addition, healthy individuals with a presumptive diagnosis of LTBI were recruited among health care workers from the Copenhagen site with a history of exposure and positive IGRA (within 2 years) without having received treatment. Healthy individuals with no known exposure to *M. tuberculosis* were recruited as controls by advertisement (forsoegsperson.dk) and enrolled at the Clinical Research Centre, Copenhagen University Hospital, Hvidovre, Denmark (Table 1). Written consent was obtained from all participants enrolled in the study.

**Table 1:** Baseline.

		Controls	TB	LTBI
**n**		96	43	13
**Age**	median (IQR)	34 (24–42)	48 (40–55)	46 (29–65)
**Male sex**	n (%)	33 (34)	29 (67)	2 (25)
**HIV status**				
Positive	n (%)	-	2 (5)	0 (0)
Negative	n (%)	-	34 (79)	10 (77)
Not done	n (%)	96 (100)	7 (16)	3 (23)
**Diagnostic assays**				
Culture and or NAAT				
Positive	n (%)	-	42 (98)	-
Negative	n (%)	-	0 (0)	-
Not done	n (%)	-	1 (2)	-
QFT-TB				
Positive	n (%)	3 (3)	26 (60)	9 (69)
Negative	n (%)	93 (97)	4 (9)	2 (15)
Not done	n (%)	0 (0)	13 (30)	2 (15)

### Whole blood stimulation and sample preparation for assay optimization

Blood was drawn in 2×10 ml Li-Hep tubes (BD Biosciences, Franklin Lakes, NJ, USA). Within 2 hours of blood draw, one of the 10 ml tubes was stimulated with 50 µl (1 mg of each peptide/ml) ESAT-6 and 50 µl (1 mg of each peptide/ml) CFP-10 peptides (18-mer peptides with 9-mer overlap, dissolved in DMSO and diluted in dH_2_0 with final concentration of each peptide of 5 µg/ml). The other 10 ml tube was stimulated with 100 µl suspension buffer (H_2_O with 37.5% DMSO). Immediately after addition of peptides, the blood was divided in 1.5 ml RNase-free Eppendorf tubes (Eppendorf, Hamburg, Germany) and incubated for up to 48 hours at 37°C with lids closed. At various time points, blood tubes were gently shaken to re-suspend cells and preparation for dried blood spots (DBS), followed by plasma isolation by centrifugation (10 min at 2000× g). DBS were made by applying 25 µl blood per spot onto Whatman FTA filter paper (Sigma-Aldrich, St. Louis, MO, USA). The spots dried at 50°C for 10 minutes after which the DBS were stored at −20°C in airtight plastic bags with desiccant until analysis.

### Whole blood stimulation for immunodiagnosis of LTBI

All patients and controls had a QFT-TB test done except 13 TB patients enrolled from Borstel and 2 LTBI individuals enrolled at Gentofte Hospital. Blood collection tubes were incubated at 37°C within 3 hours of blood draw. After 8 hours incubation, DBS samples were prepared as described in previous section. Tubes were returned to the incubator before plasma isolation at 20 hours post stimulation.

### RNA extraction from whole blood

Total RNA was extracted from 300 µl whole blood using High Pure RNA isolation kit (Roche, Schlieren, Switzerland) following manufacturers' instructions. Total RNA was eluted in 50 µl elution buffer and stored at −20°C.

### RNA extraction from dried blood spots

RNA was extracted from DBS using RNeasy mini kit (Qiagen, Hilden, Germany). Two 6 mm discs were punched from each paper sheet (Harris, Sigma-Aldrich, St. Louis, MO, USA) and discs were soaked in 350 µl RLT buffer in an RNase-free eppendorf tube (Eppendorf, Hamburg, Germany). After a brief vortex, the tube was centrifuged for 3 minutes (14,000× g) and 350 µl 70% ethanol was added and mixed by pipetting. The suspension along with the 2 DBS discs were transferred to the RNeasy spin column and centrifuged for 15 seconds (8,000× g). The two discs were carefully removed from the spin column using a pipet tip and manufacturer's protocol was followed onwards. Total RNA was eluted in 30 µl elution buffer and stored at −20°C.

### Probe based multiplex one-step RT-qPCR assay

RT-qPCR was performed with the extracted RNA as template using primers and hydrolysis probes specific for IP-10 and IFN-γ with β-actin as reference and normalization gene using the HawkZ05 Fast one-step RT-PCR kit (Roche Custom Biotech, Mannheim, Germany) as per manufacturer's protocol. A volume of 4 µl total RNA was used as template in a total reaction volume of 20 µl. Reaction mix contained a final Manganese Acetate concentration of 1.5 mM.

The primer and probe sequences and concentrations are given:

IP-10 forward: 5′-TGT CCA CGT GTT GAG ATC ATT G-3′, IP-10 reverse: 5′-GGC CTT CGA TTC TGG ATT CA-3′, 0.3 µM, 75 bp. IP-10 probe: FAM-5′-TAC AAT GAA AAA GAA GGG TGA GAA-3′-MGB, 0.2 µM [21].

IFN-γ forward: 5′-TGA ATG TCC AAC GCA AAG CA-3′. IFN-γ reverse: 5′-CGA CCT CGA AAC AGC ATC TGA-3′, 0.5 µm, 109 bp. IFN-γ probe: FAM-5′-CGC CAG CAG CTA AAA CAG GGA AGC G-3′-BHQ-1, 0.1 µM.

β-actin forward: 5′-AGC CTC GCC TTT GCC GA-3′, β-actin reverse: 5′-CTG GTG CCT GGG GCG-3′, 0.5 µM, 174 bp, β-actin probe: HEX-5′-CCG CCG CCC GTC CAC ACC CGC C-3′-BHQ-1, 0.05 µM [22].

The RT -qPCR parameters for all targets were 5 minutes at 55°C, 5 minutes at 60°C and 5 minutes at 65°C for the reverse transcription step followed by 45 cycles of 10 seconds at 94°C and 40 seconds at 56°C (LightCycler 480 II with default software, Roche, Basel, Switzerland). IP-10 and β-actin and IFN-γ and β-actin were analysed in multiplex and average Ct values were based on duplicate measurements. Primer and probe concentration and temperature optimization was performed on a Roche LightCycler 96 (Roche, Basel, Switzerland). The mRNA fold change was calculated using the 2^−ΔΔCt^ equation [23].

### Protein detection

IP-10 protein levels were determined in plasma samples using an in-house IP-10 ELISA assay in a ×30 dilution as described previously [17]. IFN-γ levels were determined using the QFT ELISA (Qiagen, Hilden, Germany) per manufacturer's instructions.

### Statistical analysis

Differences in responses were compared using Kruskal Wallis tests, diagnostic accuracy using Receiver operating characteristic (ROC) curves using GraphPad Prism 6 (GraphPad Software Inc., La Jolla, CA, USA).

## Results

### Participants

Following informed consent, 43 Patients with tuberculosis (27 from the site in Germany and 16 from the site in Denmark), 13 individuals with LTBI and 96 healthy individuals with no known exposure to *M. tuberculosis* were enrolled in the study. Forty-two of 43 TB patients (98%) had microbiologically confirmed diagnosis, one (2%) was included based on TB suspect chest X-ray changes and clinical symptoms. Patients and individuals with LTBI were significantly older than controls, and more TB patients were men (67%) compared to the other groups. Three controls had positive QFT-TB test results. Two individuals with presumptive LTBI had negative QFT-TB test results and another two were not determined.

### Validation of RT-qPCR assay for IP-10, IFN-γ and β-actin

**Figure 1:**
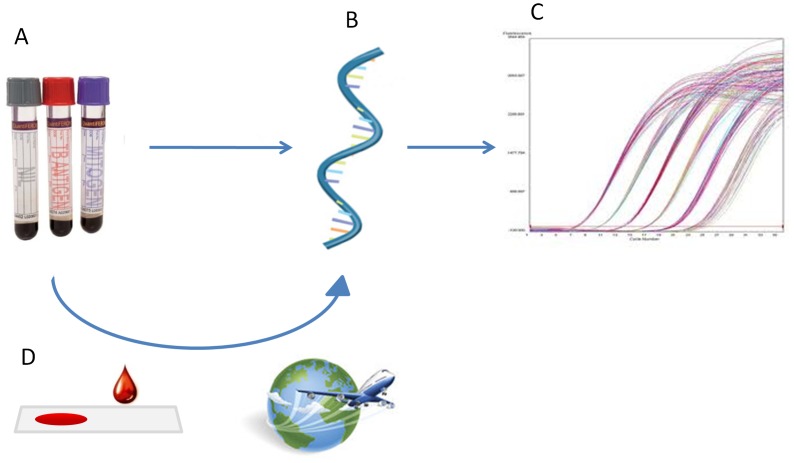
Overview of the RT-qPCR based method for immunodiagnosis of *M. tuberculosis* infection. A. Blood collection and incubation at 37C. B. Extraction of RNA. C. One-step probe based RT-qPCR and calculation of IP-10 mRNA upregulation using ΔΔCt. D. Optional RNA storage on dried blood spots for easy sample transportation.

We developed and optimized two parallel one-step RT-qPCR multiplex assays for IP-10 and IFN-γ using β-actin as reference gene (figure 1). The dynamic ranges of the assays were determined by serially diluting mRNA extracted from Phytohaemagglutinin (PHA) stimulated whole blood up to 2^13^ times. The dynamic ranges for IP-10, IFN-γ and β-actin were 22.63–34.16 Ct (r^2^ = 0.99), 22.31–34.73 Ct (r^2^ = 0.98) and 22.75–35.44 Ct (r^2^ = 0.99), respectively (figure S1). At further dilutions, we observed loss of linearity (data not shown), wherefore measurements outside the dynamic range were set to the lower limit. The PCR efficiency was calculated from the slope of the standard curve and all 3 assays showed >96% efficiency. Intra and inter assay variability and total imprecision was assessed in four representative samples analysed in quadruplicates on four consecutive days [24], and the assay was found to be very accurate (CV<1.15% (table S1)). A panel of six reference genes were tested from which β-actin was selected as the gene least affected by antigen and PHA mitogen stimulation (data not shown).

### Whole blood vs. dried blood spot

**Figure 2:**
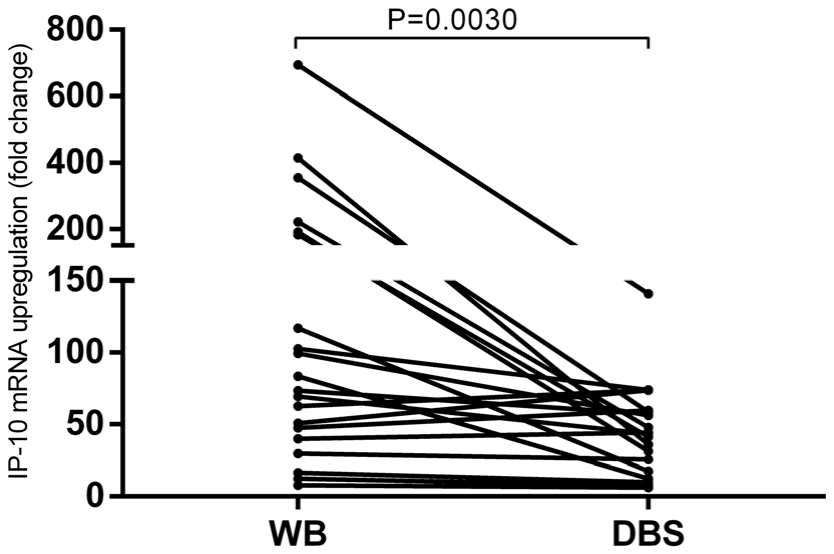
Comparison of mRNA extraction from whole blood (WB) and dried blood spots (DBS). Whole blood from 12 TB patients and 8 LTBI persons was incubated in QFT-TB tubes for 8 hours at 37°C. mRNA was extracted directly from WB and DBS samples were prepared for later mRNA extraction. IP-10 gene expression was determined using our RT-qPCR assay. The difference was analysed using a Wilcoxon matched pairs test *p* = 0.003.

After establishing and validating the RT-qPCR assays, we explored the possibility of determining mRNA expression from dried blood spots (DBS). Whole blood from 20 donors with known QFT-TB positivity was stimulated with *M. tuberculosis* antigens for 8 hours after which mRNA was extracted directly from whole blood or from DBS samples (figure 2). Fold change measurements were significantly higher in whole blood (median 78.3, IQR 41.8–188.1) compared to DBS samples (median 43.9, IQR 19.5–59.4) (*p* = 0.003), median fold change ratio between WB and DBS was 1.7 (IQR 1.2–5.8). After demonstrating feasibility of mRNA extraction from DBS, stability was assessed of mRNA in DBS samples in three representative samples stored at up to 50°C for 28 days in double sealed zip lock bags with desiccant. No indication of mRNA degradation was observed even at high temperatures (Figure S2) thus demonstrating that the DBS and RT-qPCR based IP-10 release assays is feasible and reliable.

### IP-10 and IFN-γ expression profile

**Figure 3:**
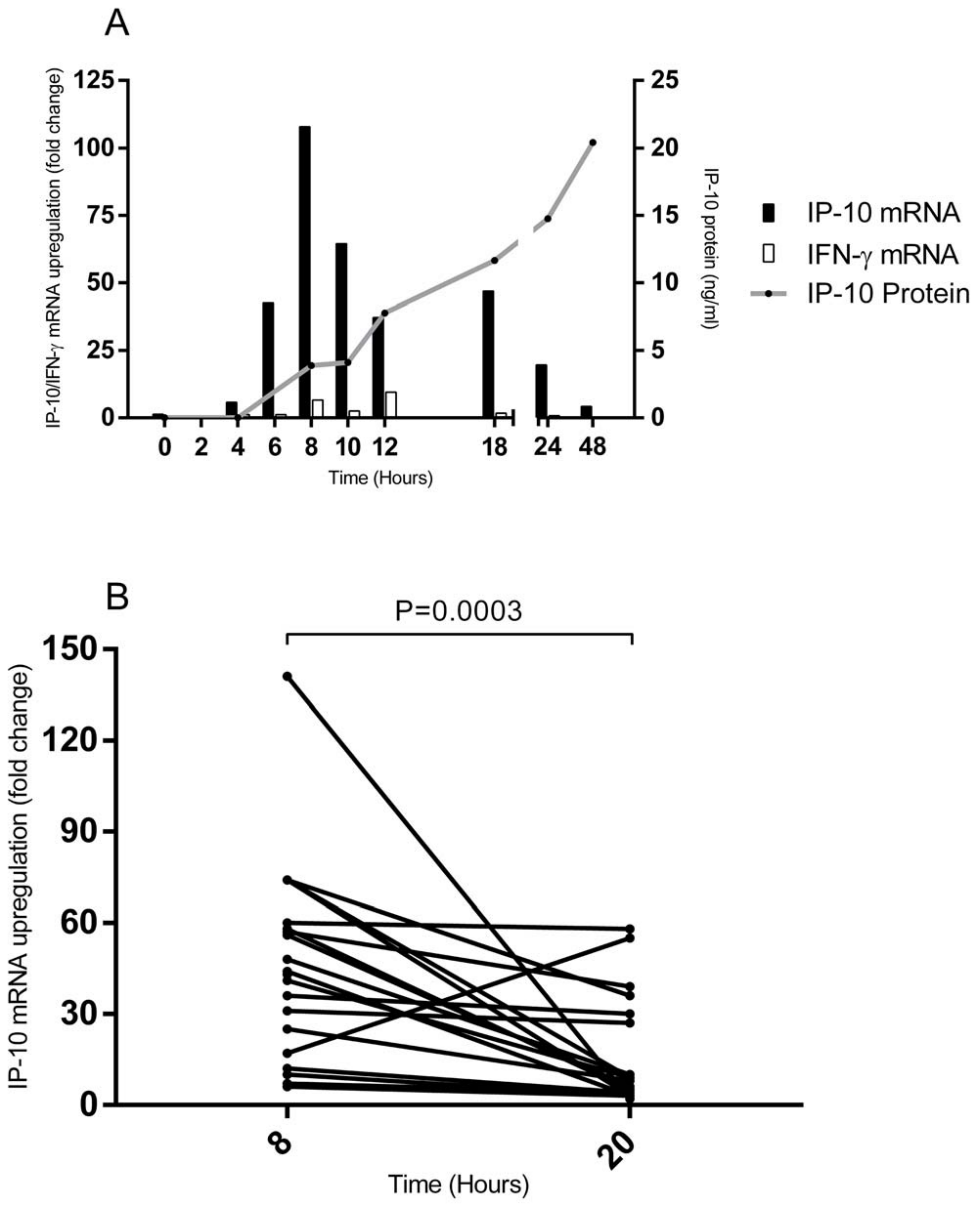
IP-10 and IFN-γ expression profiles. A: Whole blood from two TB patients and two persons with known QFT-TB positivity was incubated in QFT-TB tubes for up to 48 hours at 37°C. Every second hour for 12 hours and at 18, 24 and 48 hours post stimulation, dried blood spots were prepared for later mRNA extraction and plasma was isolated for protein analysis except for 2, 4 and 6 hours post stimulation. IP-10 and IFN-γ gene expression specified as mRNA fold change was determined using our RT-qPCR assay and IP-10 protein levels were determined using an in-house IP-10 ELISA assay. The black bars represent median IP-10 mRNA upregulation in fold change, the white bars represent the IFN-γ mRNA upregulation and the grey line represents the median IP-10 protein expression. IFN-γ protein expression was not measured in this experiment. B: Whole blood from 12 TB patients and 8 LTBI persons was incubated in QFT-TB tubes for up to 20 hours at 37°C. Dried blood spots were made after 8 hours incubation and after 20 hours incubation. mRNA was subsequently extracted and IP-10 mRNA fold change was determined using our RT-qPCR assay. The difference was analysed using a Wilcoxon matched pairs test *p = 0.0003*.

The simple sample preparation of the DBS RT-qPCR assay enabled a detailed exploration of the interplay of IP-10 and IFN-γ genes and IP-10 protein expression kinetics in whole blood cultures. We observed detectable IP-10 gene expression at 4 hours (approx. 6 fold) and peak levels at 8 hours with levels declining hereafter. IP-10 protein expression described a more protracted secretion profile reaching plateau approximately 24 hours post stimulation. The IFN-γ mRNA expression profile followed IP-10 but levels were lower (IFN-γ median 6.7 fold, IQR 2.8–12.1 at 8 hours and IP-10 median 108.0 fold, IQR 47.4–243.3 at 8 hours, p<0.0001) (figure 3A). The data point for the individual donors together with the standard deviations can be seen in Table S2. The superiority of 8 hours incubation was confirmed in a separate cohort of 12 tuberculosis patients and 8 QFT-TB test positive persons comparing IP-10 mRNA upregulation at 8 hours and 20 hours (figure 3B).

### Diagnostic potential for IP-10 RT-qPCR assay

**Figure 4:**
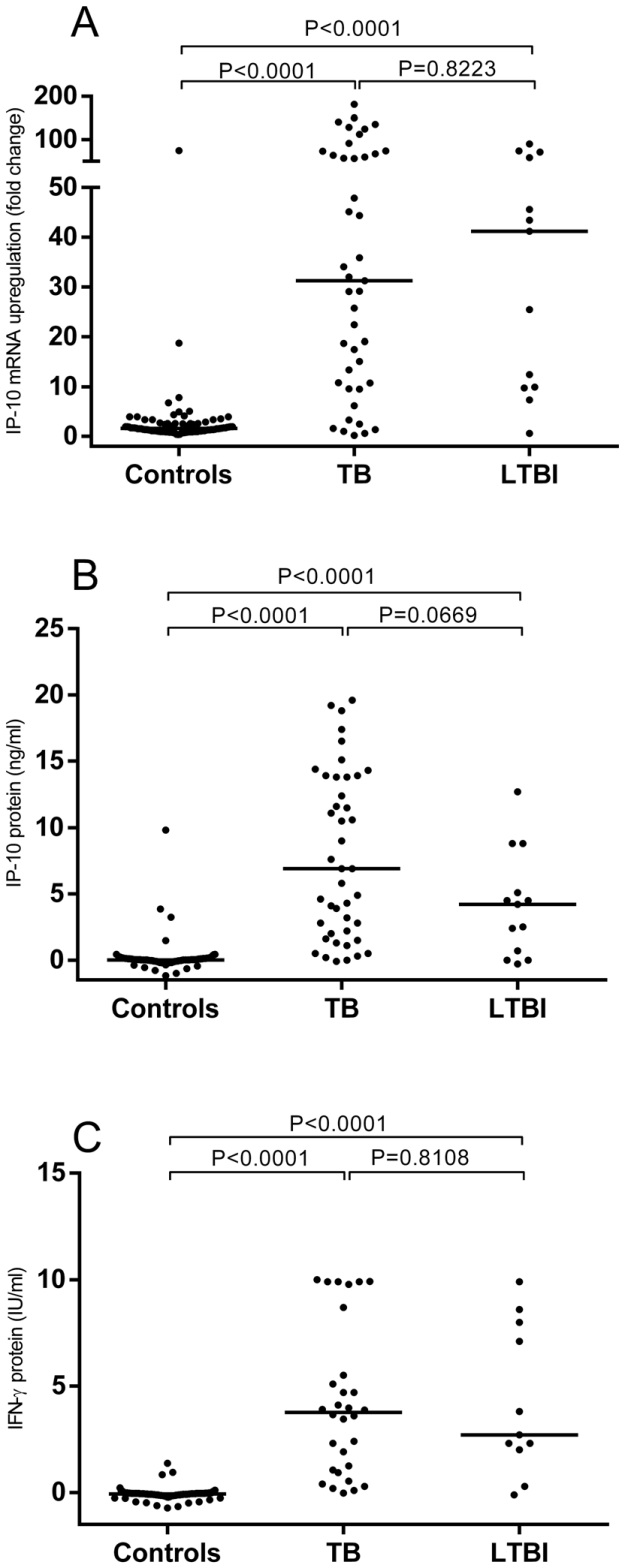
IP-10 mRNA expression and IP-10 and IFN-γ protein release. Whole blood from 96 healthy controls, 43 TB patients and 13 LTBI persons was stimulated with ESAT-6, CFP-10 and TB7.7. IP-10 gene expression was analysed from DBS after 8 hours stimulation (A) and IP-10 and IFN-γ protein levels were analysed from plasma after 20 hours stimulation (B and C respectively). A Kruskal Wallis test was performed to analyse the differences between the groups. IFN-γ mRNA gene expression was not measured in this experiment.

We assessed the diagnostic potential of the DBS based IP-10 RT-qPCR assay in 96 presumed healthy controls, 43 culture confirmed TB patients and 13 persons with LTBI. All samples were measured in standard QFT blood collection tubes. IP-10 gene expression levels were significantly higher in patients with tuberculosis (median 31.2, IQR 10.7–67.0) and persons with LTBI (41.2, IQR 9.8–64.9) compared to healthy controls (1.6, IQR 1.1–2.4) (figure 4A). A similar pattern was found for IP-10 protein expression with tuberculosis patients (median 6.9 ng/ml, IQR 2.0–13.8), persons with LTBI (median 4.2 ng/ml, IQR 0.4–7.0) and controls (median (0.0 ng/ml, IQR 0–0.1) (figure 4B). IFN-γ protein expression followed a similar pattern, where tuberculosis patients (median 3.8 IU/ml, IQR 1.0–6.3) and persons with LTBI (median 2.7 IU/ml, IQR 2.0–8.0) had higher levels compared to controls (median 0.0 IU/ml, IQR 0.0–0.0) (figure 4C).

### ROC Curve analysis

**Figure 5:**
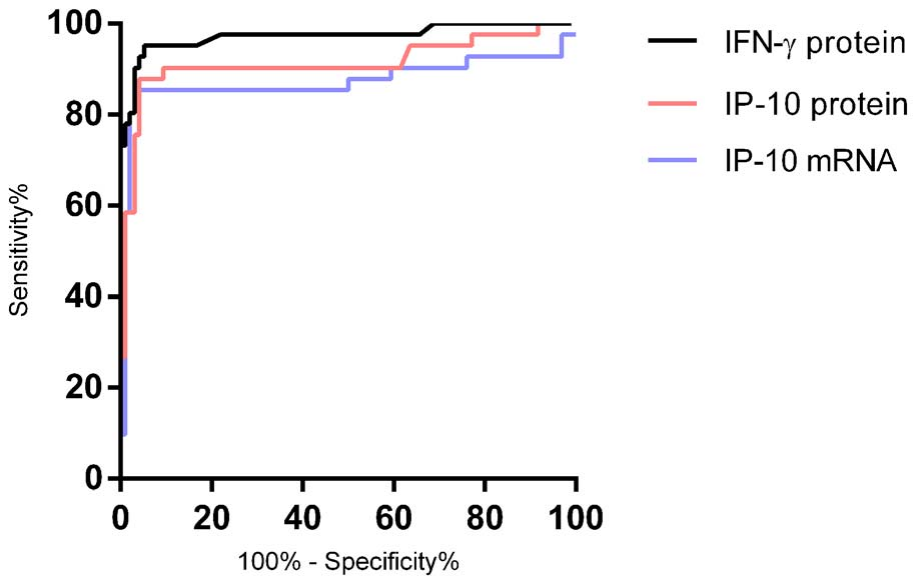
Comparison of the diagnostic potential of IP-10 RT-qPCR, IP-10 protein and IFN-γ protein. ROC curve comparison of antigen-specific IP-10 mRNA expression and IP-10 and IFN-γ protein release. Cases comprised 30 TB patients and 13 LTBI persons and controls were 96 persons with no known exposure to *M. tuberculosis*. IP-10 gene expression was analysed from DBS and IP-10 and IFN-γ protein levels were analysed in plasma samples. AUCs was comparable at 0.88, 0.91 and 0.97 for IP-10 mRNA, IP-10 protein and QFT-TB respectively (*p*<0.0001). Cut offs were selected at the point rendering high sensitivity without compromised specificity.

We compared the diagnostic potential of the RT-qPCR assay head to head with IP-10 and IFN-γ determined at the protein level. The IP-10 DBS based mRNA and plasma based protein tests were comparable with AUCs of 0.87 and 0.91, suggesting cut-off values of 5.6 fold change (sensitivity 85%, specificity 96%) and 0.47 ng/ml (sensitivity 88%, specificity 96%), respectively (figure 5). The AUC of IFN-γ was 0.97, but after applying the manufacturer's cut-off (0.35 IU/ml), the sensitivity and specificity was comparable to IP-10 (85% and 97%) thus underpinning that the differences in AUC between IP-10 and IFN-γ is driven by a small group of patients with IFN-γ responses below the cut off.

## Discussion

In this study, we describe the development of an accurate novel molecular test using IP-10 mRNA for the diagnosis of *M. tuberculosis* infection from dried blood spots. This test has comparable diagnostic accuracy to the commercially available QFT-TB test and provides substantial operational advantages, especially for the diagnosis of *M. tuberculosis* infections in remote settings.

### Molecular immunodiagnostics

Molecular assays are attractive as diagnostic tests due to high analytical accuracy, rapidity and suitability for fully automated workflows. For immunodiagnostics in particular, mRNA-based tests are not affected by the pre-existing cytokine level in the blood wherefore the risk of indeterminate results due to high nil is eliminated. Also, as mRNA expression inevitably precedes protein release, molecular immunodiagnostics require shorter incubation time compared to conventional protein based tests, a reduction from 16–24 hours possibly to as little as 4 hours.

Wu et al. were the first to demonstrate feasibility of molecular immunodiagnosis of *M. tuberculosis* infection [25]. Using an ambitious 45 cytokine plex mRNA based approach, PBMCs were stimulated with ESAT-6/CFP10 and mRNA was extracted after 15 hours. Bibova extended the technology to chemokine-based assays and later – and in more detail - Kasprovicz combined IP-10 and MIG in a SYBR-Green based RT-qPCR assay from whole blood stimulated overnight [19], [26]. Kim et al, determined the kinetics of IFN-γ mRNA release in response to ESAT-6 and CFP10 stimulation, and established that antigen specific IFN-γ gene expression peaks at 6–10 hours after stimulation [20]. Using our one-step probe based RT-qPCR approach, we confirm these results and further explored the interplay of IFN-γ and IP-10 as markers in immunodiagnostic assays. IP-10 expression was very high and often >100 fold upregulated compared to the unstimulated sample: In comparison IFN-γ expression was 16 fold lower and less consistent, wherefore we did not include IFN-γ mRNA in the later experiments. The dramatic IP-10 mRNA responses detected after 8 hours, strongly suggest that this assay is very sensitive and potentially able to detect responses either from few cells or upon stimulation with subdominant antigens. Such highly sensitive assays are important in e.g. vaccine trials where measurements of immunogenicity at late time points rely on small populations of specific cells and when screening for viral epitopes [27]–[29].

Somewhat to our surprise, IP-10 and IFN-γ gene expression occurs in concert. This indicates that IP-10 gene expression in the antigen presenting cell happens very early on in adaptive immune responses, and that these early events occur - at least in part – independently of IFN-γ secretion and possibly also the phenotype of the antigen-specific T cell. If this hypothesis can be confirmed, it could explain the paradoxical observations of very high IP-10 protein release occurring in some whole blood cultures with low or absent IFN-γ release, and it could be the underlying mechanism driving the superior sensitivity of IP-10 in HIV infected TB patients with relative IFN-γ anergy [30]–[33].

IP-10 mRNA expression is an early event underpinning the importance of timing of the mRNA purification step. Stabilizing mRNA using the DBS method is a simple approach well suitable for field use. In this study, we also assessed if storage at +5°C for an additional 12 hours can prevent mRNA degradation and found no significant loss of signal compared to immediate mRNA extraction (data not shown). Alternative and more laboratory friendly approaches include mRNA stabilization by addition of RNALater (Ambion, Austin TX, USA) or PAXgene (BD Biosciences, Franklin Lakes, NJ, USA) [29].

Interestingly, as IP-10 mRNA levels were clearly detectable at 4 hours it seems feasible that an automated IP-10 based molecular immunodiagnostic test can deliver results within 6 hours including sample incubation, sample processing and reporting. The combination of a highly specific probe based one-step RT-qPCR assay and a highly expressed mRNA target ensures optimal test performance. The lower signal-to-noise ratio seen with IFN-γ, IL-2 and other cytokines, renders these less attractive markers from a technical point of view. However, the increased sensitivity offered by the RT-qPCR method does suggest that alternative cytokines, which have been associated with *M. tuberculosis* infection control, could be detected more accurately with this method [12], [34].

### IP-10 expression kinetics, an association with risk of disease?

An avenue for further research is to explore the association between IP-10 expression kinetics and risk of progression to tuberculosis. It is established, that the T cell phenotype in patients with ongoing bacterial replication is dominated by effector memory cells in contrast to individuals with controlled infection and low bacterial replication that have a dominance of central memory T cell [35]. Therefore, it is tempting to speculate that the time to detectable IP-10 gene expression is associated with the degree of ongoing anti-mycobacterial immune activity and herewith incipient disease [36]. A test that can pinpoint the individuals with highest risk of disease within a group of IGRA positive will have tremendous impact on the management of individuals at risk of tuberculosis and warrants exploration.

### mRNA extraction from dried blood spots

A major limitation to the IGRAs is the labour intensive and instrument dependent steps required when measuring IFN-γ release. As this is done using live cells or in potentially infectious plasma samples, the laboratory work must be done close to where blood is drawn. Reduced requirements for skilled staff and laboratory facilities would reduce costs and enable specific immunodiagnostics in remote settings. Recently, we described an IP-10 release assay based on IP-10 protein extracted from both DBS and dried plasma spots [17]. We validated this assay in clinical cohorts and demonstrated diagnostic accuracy at par with IGRA and IP-10 detected from plasma and demonstrated that DBS samples can be sent across Europe by normal mail before analysis with no loss of diagnostic accuracy [30], [37]. Inspired by these activities we attempted mRNA extraction from DBS. DBS technology is a simple and reliable method for storage of proteins and genomic material [38], [39] and has been the cornerstone in screening programs for inherited metabolic conditions in neonates since the 1960's [40]. In contrast to the fragility of mRNA molecules in solution, mRNA appears very robust in dried form. This was clearly demonstrated by successful extraction of mRNA from DBS samples stored for >20 years at ambient temperatures [38], [40], [41], and our findings of no loss of mRNA signal after storage for up to 50°C for at least 28 days (Figure S2).

We have shown proof of concept for this molecular assay using IP-10 mRNA extraction from DBS. DBS yields 1.7 times lower fold change values compared to extraction from whole blood and is as such more difficult and inferior compared to mRNA extracted directly from whole blood. Furthermore, the small sample volume retained in DBS (≈50 µl blood) renders RNA concentration below detection limit of even sensitive spectrophotometers such as the NanoDrop 1000 (data not shown) which makes standardisation of the RNA template input concentration in the RT-qPCR assay impossible. Thus, for our DBS based assay we assume the extraction efficiency to be constant, an assumption we are comfortable with as all calculated fold changes in the DBS stability test was within range of the expected variability of the RT-qPCR assay (figure S2).

## Conclusions

In conclusion, we developed a probe based one-step multiplex RT-qPCR assay for whole blood and DBS samples with high PCR efficiency (>96%) and high reproducibility (CV<1.15%). We showed that the diagnostic potential of the DBS based assay was comparable to that of the commercially available QFT-TB test. By combining DBS based sample acquisition, mail or currier based sample transport with centralized molecular detection, this immunodiagnostic test concept will reduce the local technological requirements thus making highly accurate immunodiagnostic tests accessible in low resource settings.

## Supporting Information

Figure S1 **Dynamic ranges of IP-10, ACTB and IFN-γ in the RT-qPCR assay.** The dynamic range of the assay was evaluated using whole blood stimulated with PHA (37.5 µg/ml) for two hours at 37°C. Total RNA was extracted from whole blood as described in materials and methods. Total RNA concentration could not be accurately evaluated as the levels were close to the detection limit of the NanoDrop 1000 (2 ng/µl). mRNA was serially diluted to ×2^13^ and each point was analysed in duplicates. A linear regression analysis was done and the PCR efficiency was calculated using PCR Efficiency (%) = (2^−1/slope^−1)×100. The calculated efficiency and r^2^ for the 3 targets are 96% (r^2^ = 0.99), 98% (r^2^ = 0.98) and 99% (r^2^ = 0.99) for IP-10, β-actin and IFN-γ respectively. Results are given with standard deviations.
(TIF)

Figure S2 **mRNA stability in Dried blood spots.** Whole blood from three healthy donors were stimulated with PHA (37.5 µg/ml). After 2 hours incubation at 37°C, donor 1 was left undiluted (A), donor 2 was diluted ×8 in unstimulated whole blood (B) and donor 3 was diluted ×64 in unstimulated whole blood (C) to obtain Ct values spanning the middle to lower part of the dynamic range of the assay. Dried blood spots were done as described in materials and methods. The DBS were stored for up to 28 days at 4°, 20°, 37° and 50°C. At days 7, 14 and 28 the DBS were stored at −20°C. Total RNA was extracted and Ct values was analysed using our IP-10 RT-qPCR assay. The samples were analysed in duplicates and results are given with standard deviations.
(TIF)

Table S1 **Total imprecision and reproducibility of one-step RT-qPCR.** The total imprecision was calculated according to Krouwer and Rabinowitz (REF). Whole blood from 3 healthy donors was stimulated with PHA (37.5 µg/ml) and total RNA was extracted after two hours of incubation at 37°C. After a preanalysis to determine the Ct value of undiluted RNA samples, the individual RNA concentrations were diluted to span the dynamic range of the assay and to obtain a total volume to perform analysis in quadruplicates in four consecutive days. Sample 1 and 4 are from the same donor however at different RNA dilutions.
(DOCX)

Table S2 **IP-10 and IFN-γ mRNA upregulations and IP-10 protein expression in individual donors in expression profile analysis.** IP-10 mRNA upregulation was analysed in duplicates and IFN-g in singlets. The data provided is the calculated mRNA upregulation in fold change using the ΔΔCt equation. The IP-10 protein is analysed in duplicates.
(DOCX)
