# Supplementary figures and images for: Development of a One-Step Probe Based Molecular Assay for Rapid Immunodiagnosis of Infection with M. tuberculosis Using Dried Blood Spots
Source: PLoS One. 2014 Sep 3;9(9):e105628. doi: 10.1371/journal.pone.0105628 (PMC4153573; doi:10.1371/journal.pone.0105628)

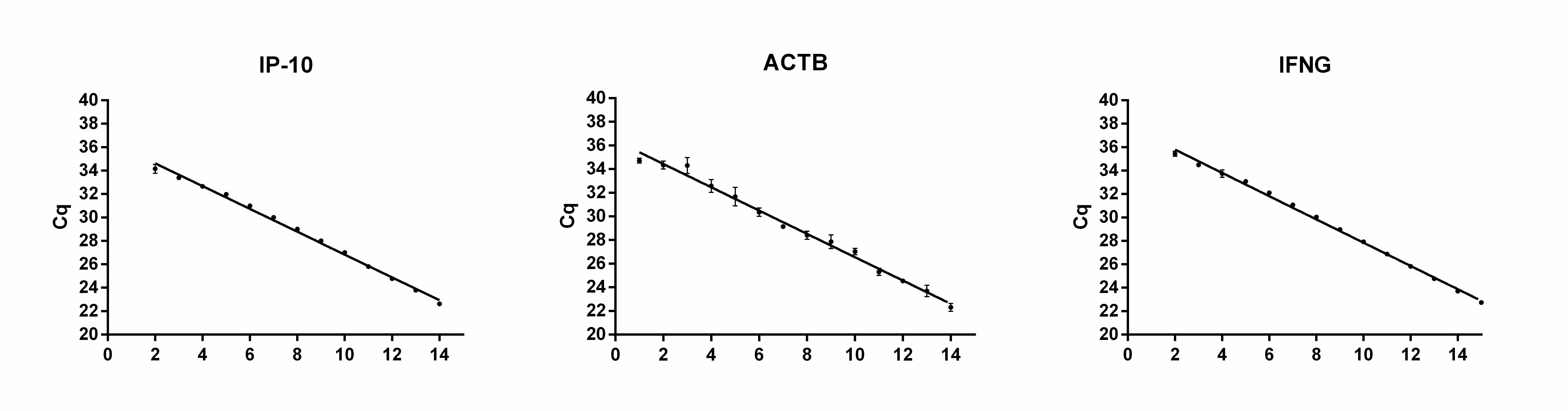

Supplement: Figure S1 — Dynamic ranges of IP-10, ACTB and IFN-γ in the RT-qPCR assay. The dynamic range of the assay was evaluated using whole blood stimulated with PHA (37.5 µg/ml) for two hours at 37°C. Total RNA was extracted from whole blood as described in materials and methods. Total RNA concentration could not be accurately evaluated as the levels were close to the detection limit of the NanoDrop 1000 (2 ng/µl). mRNA was serially diluted to ×213 and each point was analysed in duplicates. A linear regression analysis was done and the PCR efficiency was calculated using PCR Efficiency (%) = (2−1/slope−1)×100. The calculated efficiency and r2 for the 3 targets are 96% (r2 = 0.99), 98% (r2 = 0.98) and 99% (r2 = 0.99) for IP-10, β-actin and IFN-γ respectively. Results are given with standard deviations. (TIF) [file pone.0105628.s001.tif]
