# Supplementary material for: Development of a One-Step Probe Based Molecular Assay for Rapid Immunodiagnosis of Infection with M. tuberculosis Using Dried Blood Spots
Source: PLoS One. 2014 Sep 3;9(9):e105628. doi: 10.1371/journal.pone.0105628 (PMC4153573; doi:10.1371/journal.pone.0105628)
Supplement: Table S1 — Total imprecision and reproducibility of one-step RT-qPCR. The total imprecision was calculated according to Krouwer and Rabinowitz (REF). Whole blood from 3 healthy donors was stimulated with PHA (37.5 µg/ml) and total RNA was extracted after two hours of incubation at 37°C. After a preanalysis to determine the Ct value of undiluted RNA samples, the individual RNA concentrations were diluted to span the dynamic range of the assay and to obtain a total volume to perform analysis in quadruplicates in four consecutive days. Sample 1 and 4 are from the same donor however at different RNA dilutions. (DOCX) [file pone.0105628.s003.docx]

Table S1. Total imprecision and reproducibility of one-step RT-qPCR

|  | **Total mean** | **Within run** | | **Between run** | | **Total Imprecision** | |
| --- | --- | --- | --- | --- | --- | --- | --- |
|  | **(Ct)** | **SD** | **CV%** | **SD** | **CV%** | **SD** | **CV%** |
| **Sample 1** | 24.96 | 0.08 | 0.33% | 0.08 | 0.31% | 0.11 | 0.45% |
| **Sample 2** | 26.35 | 0.14 | 0.54% | 0.17 | 0.65% | 0.22 | 0.85% |
| **Sample 3** | 29.13 | 0.16 | 0.55% | 0.29 | 1.01% | 0.33 | 1.15% |
| **Sample 4** | 31.89 | 0.15 | 0.46% | 0.00 | 0.00% | 0.15 | 0.46% |

The total imprecision was calculated according to Krouwer and Rabinowitz (REF). Whole blood from 3 healthy donors was stimulated with PHA (37.5µg/ml) and total RNA was extracted after two hours of incubation at 37°C. After a preanalysis to determine the Ct value of undiluted RNA samples, the individual RNA concentrations were diluted to span the dynamic range of the assay and to obtain a total volume to perform analysis in quadruplicates in four consecutive days. Sample 1 and 4 are from the same donor however at different RNA dilutions.
