# Supplementary material for: Development of a One-Step Probe Based Molecular Assay for Rapid Immunodiagnosis of Infection with M. tuberculosis Using Dried Blood Spots
Source: PLoS One. 2014 Sep 3;9(9):e105628. doi: 10.1371/journal.pone.0105628 (PMC4153573; doi:10.1371/journal.pone.0105628)
Supplement: Table S2 — IP-10 and IFN-γ mRNA upregulations and IP-10 protein expression in individual donors in expression profile analysis. IP-10 mRNA upregulation was analysed in duplicates and IFN-g in singlets. The data provided is the calculated mRNA upregulation in fold change using the ΔΔCt equation. The IP-10 protein is analysed in duplicates. (DOCX) [file pone.0105628.s004.docx]

Table S2 IP-10 and IFN-g mRNA upregulations and IP-10 protein expression in individual donors in expression profile analysis

|  | **Time (hours)** | **0** | | **2** | | **4** | | **6** | | | **8** | | | **10** | | | **12** | | | **18** | | **24** | | **48** | |
| --- | --- | --- | --- | --- | --- | --- | --- | --- | --- | --- | --- | --- | --- | --- | --- | --- | --- | --- | --- | --- | --- | --- | --- | --- | --- |
|  |  | **Ave**  **rage** | **Std dev.** | **Ave**  **rage** | **Std dev.** | **Ave**  **rage** | **Std dev.** | **Ave**  **rage** | | **Std dev.** | **Ave**  **rage** | | **Std dev.** | **Ave**  **rage** | | **Std dev.** | **Ave**  **rage** | | **Std dev.** | **Ave**  **rage** | **Std dev.** | **Ave**  **rage** | **Std dev.** | **Ave**  **rage** | **Std dev.** |
| **IP-10 mRNA (fold change)** | **Donor 1** | 4.1 | 0.0 | 1.0 | 0.5 | 4.6 | 0.2 | 19.3 | | 2.5 | 138.7 | | 4.1 | 124.3 | | 11.0 | 39.6 | | 5.2 | 112.7 | 14.3 |  |  |  |  |
|  | **Donor 2** | 0.7 | 0.1 | 0.5 | 0.1 | 24.3 | 0.5 | 121.9 | | 0.0 | 36.9 | | 1.8 | 34.3 | | 3.7 | 35.4 | | 4.5 | 8.6 | 1.6 | 19.6 | 0.3 | 4.3 | 0.6 |
|  | **Donor 3** | 1.6 | 0.0 | 1.8 | 0.1 | 1.0 | 0.0 | 44.4 | | 0.6 | 290.3 | | 18.5 | 48.4 | | 2.3 | 111.8 | | 13.2 | 67.9 | 3.0 | 47.6 | 2.3 | 3.1 | 0.4 |
|  | **Donor 4** | 0.9 | 0.0 | 0.3 | 0.1 | 7.2 | 0.5 | 39.6 | | 2.3 | 77.5 | | 3.8 | 88.6 | | 13.4 | 3.3 | | 0.1 | 27.8 | 0.4 | 14.5 | 1.4 | 10.8 | 0.3 |
|  |  |  |  |  |  |  |  |  | |  |  | |  |  | |  |  | |  |  |  |  |  |  |  |
|  | **Time (hours)** | **0** | **2** | **4** | **6** | **8** | **10** | **12** | | **18** | **24** | | **48** |  | |  |  | |  |  |  |  |  |  |  |
| **IFN-γ mRNA (fold change)** | **Donor 1** | 1 | 0.6 | 0.6 | 1.2 | 5.2 | 2.8 | 4.1 | | 2.5 |  | |  |  | |  |  | |  |  |  |  |  |  |  |
|  | **Donor 2** | 0.8 | 0.3 | 1.4 | 6.5 | 8.1 | 7.1 | 3.4 | | 2.1 | 1.9 | | 0.2 |  | |  |  | |  |  |  |  |  |  |  |
|  | **Donor 3** | 0.4 | 0.6 | 1 | 0.1 | 13.4 | 0.6 | 28.2 | | 0.7 | 0.8 | | 0.1 |  | |  |  | |  |  |  |  |  |  |  |
|  | **Donor 4** | 0.6 | 0.9 | 1.6 | 1.1 | 2 | 2.2 | 15 | | 1.3 | 0.5 | | 0.1 |  | |  |  | |  |  |  |  |  |  |  |
|  |  |  |  |  |  |  |  |  | |  |  | |  |  | |  |  | |  |  |  |  |  |  |  |
|  | **Time (hours)** | **0** | | **4** | | **8** | | **12** | | | **18** | | | **24** | | | **48** | | |  |  |  |  |  |  |
|  |  | **Average** | **Std dev.** | **Ave**  **rage** | **Std dev.** | **Ave**  **rage** | **Std dev.** | **Ave**  **rage** | **Std dev.** | | **Ave**  **rage** | **Std dev.** | | **Ave**  **rage** | **Std dev.** | | **Ave**  **rage** | **Std dev.** | |  |  |  |  |  |  |
| **IP-10 Protein (ng/ml)** | **Donor 1** | 0.0 | 0.0 | 0.0 | 0.0 | 2.3 | 0.0 | 7.3 | 0.3 | | 11.2 | 0.1 | | 15.1 | 0.4 | | 22.5 | 0.4 | |  |  |  |  |  |  |
|  | **Donor 2** | 0.0 | 0.0 |  |  | 10.7 | 0.0 | 28.8 | 0.7 | | 35.9 | 0.9 | | 43.8 | 1.2 | | 47.5 | 1.4 | |  |  |  |  |  |  |
|  | **Donor 3** | 0.0 | 0.0 |  |  | 10.7 | 0.0 | 28.8 | 0.7 | | 35.9 | 0.9 | | 43.8 | 1.2 | | 47.5 | 1.4 | |  |  |  |  |  |  |
|  | **Donor 4** | 0.2 | 0.0 |  |  | 3.3 | 0.0 | 7.7 | 0.2 | | 11.9 | 0.2 | | 14.2 | 0.8 | | 10.8 | 0.7 | |  |  |  |  |  |  |

IP-10 mRNA upregulation was analysed in duplicates and IFN-g in singlets. The data provided is the calculated mRNA upregulation in fold change using the ΔΔCt equation. The IP-10 protein is analysed in duplicates.
